# Stabilization of *Arabidopsis* DREB2A Is Required but Not Sufficient for the Induction of Target Genes under Conditions of Stress

**DOI:** 10.1371/journal.pone.0080457

**Published:** 2013-12-23

**Authors:** Kyoko Morimoto, Junya Mizoi, Feng Qin, June-Sik Kim, Hikaru Sato, Yuriko Osakabe, Kazuo Shinozaki, Kazuko Yamaguchi-Shinozaki

**Affiliations:** 1 Laboratory of Plant Molecular Physiology, Graduate School of Agricultural and Life Sciences, The University of Tokyo, Bunkyo-ku, Tokyo, Japan; 2 Biological Resources and Post-Harvest Division, Japan International Research Center for Agricultural Sciences, Tsukuba, Japan; 3 Research Institute for Agriculture and Life Sciences, Seoul National University, Seoul, Korea; 4 RIKEN Center for Sustainable Resource Science, Yokohama, Japan; University of Delhi South Campus, India

## Abstract

The *Arabidopsis thaliana* transcription factor DEHYDRATION-RESPONSIVE ELEMENT-BINDING PROTEIN2A (DREB2A) controls the expression of many genes involved in the plant's response to dehydration and heat stress. Despite the significance of post-translational regulation in DREB2A activation, the mechanism underlying this activation remains unclear. Here, with the aid of a newly produced antibody against DREB2A, we characterized the regulation of DREB2A stability in plants exposed to stress stimuli. Endogenous DREB2A accumulated in wild-type *Arabidopsis* plants subjected to dehydration and heat stress. A degradation assay using *Arabidopsis* T87 suspension-cultured cells revealed that DREB2A protein degradation was inhibited at high temperatures. The proteasome-dependent degradation of DREB2A required the import of this protein into the nucleus. The E3 ligases DRIP1 and DRIP2 were involved in this process under both normal and stressful conditions; however, other E3 ligases may have also been involved, at least during the late stages of the heat stress response. Although the constitutive expression of *DREB2A* resulted in an overproduction of DREB2A and enhanced target gene induction during stress in transgenic plants, the accumulation of DREB2A caused by proteasome inhibitors did not induce target gene expression. Thus, the stabilization of DREB2A is important but not sufficient to induce target gene expression; further activation processes are required.

## Introduction

Plants are often exposed to environmental stress, such as drought, high salinity and extreme temperatures and have developed a number of elaborate mechanisms to respond and adapt to these adverse environmental conditions. Transcriptional modulation is one of the most important mechanisms utilized by plants to respond and adapt to stress. Extensive analyses of stress-responsive genes revealed that a variety of transcription factors are involved in signal transduction network, from the perception of stress signals to the expression of stress-responsive genes related to stress tolerance and growth regulation [1]–[4]. Post-transcriptional mechanisms based on alternative splicing, RNA processing and RNA silencing are also involved in abiotic stress responses, as early reported [5]–[6]. Additionally, the post-translational regulation of transcription factors in this network via phosphorylation, ubiquitination and sumoylation is believed to ensure prompt responses to stresses [7]–[11].

DEHYDRATION-RESPONSIVE ELEMENT-BINDING PROTEIN2A (DREB2A) of *Arabidopsis thaliana* is a key transcription factor involved in the signal transduction network that controls the plant's response to dehydration and heat stress. DREB2A is an ethylene-responsive element binding factor/APETALA2 (ERF/AP2) family transcription factor, and governs the expression of many stress-inducible target genes via a specific *cis*-acting element, the dehydration responsive element/C-repeat (DRE/CRT; A/GCCGAC) [12], [13]. The expression of the *DREB2A* gene is induced by dehydration or heat shock via independent *cis*-acting elements in its promoter region [14], [15]. However, the ectopic overexpression of *DREB2A* does not effectively activate the transcription of target genes because of a post-translational negative regulatory system [16]–[18]. The post-translational regulation of DREB2A involves a Ser- and Thr-rich 30-amino acid region termed as NRD (negative regulation domain) in the middle of the protein [16]. The removal of the NRD yields a constitutively active form of DREB2A (DREB2A CA). A GFP-DREB2A CA fusion protein exhibited stronger fluorescence in the nucleus than the wild-type protein under normal conditions, indicating that DREB2A CA is more stable than the wild-type protein. The overexpression of DREB2A CA induced target gene expression in transgenic plants (even under non-stressful conditions) and enhanced the plant's ability to tolerate dehydration and heat stress [16], [17]. The overexpression of DREB2A CA also negatively affected plant growth; transgenic plants exhibited dwarfism and a compromised ability to reproduce, and the severity of the phenotypes was correlated with levels of DREB2A CA expression.

DREB2A-INTERACTING PROTEIN1 (DRIP1) and DRIP2, which are C3HC4 RING domain-containing proteins, were identified as DREB2A interactors that function as E3 ubiquitin ligases in the nucleus and act as negative regulators in stress-responsive gene expression by targeting DREB2A to 26S proteasome-mediated proteolysis [18]. A GFP-DREB2A fusion protein expressed under the native promoter accumulated at high levels in the nucleus in response to heat, which implies that the activation of DREB2A coincides with its stabilization (*i.e.*, inhibition of targeted proteolysis) [17]. However, it remains unclear whether this stabilization is the sole determinant of DREB2A activation. To address this question, we investigated the relationship between the accumulation of DREB2A and the expression of its target genes. Our findings indicate that, although the amount of DREB2A affects the strength of target expression, the stabilization of DREB2A is not sufficient for its full transcriptional activity; thus, further activation may be required.

## Materials and Methods

### Plant materials, growth conditions and transformation

*Arabidopsis thaliana* (L.) Heynh. ecotype Columbia was used as the wild-type (WT) line. The *Arabidopsis* T-DNA insertion lines *dreb2a1*, *drip1* and *drip1 drip2* were described previously [17], [18]. *Arabidopsis* plants were grown on germination medium (GM) agar plates at 22°C under a day/night light regime with a 16-h photoperiod at a photon density of 40±10 µmol photons m^−2^ s^−1^ [13] and transformed as previously described [14]. The *Arabidopsis* suspension-cultured cell line T87 [19] was maintained and transformed according to the method described in a previous report [20]. Transient protein expression in *Nicotiana benthamiana* plants was performed as previously described [21]. Detailed procedures describing the construction of plasmids for plant transformation are provided in Methods S1.

### Abiotic stress and chemical treatments

For the dehydration stress treatment, three-week-old *Arabidopsis* seedlings were removed from the GM agar plates and placed on a piece of Parafilm in empty Petri dishes under dim light conditions on a clean bench at 22±2°C. For the heat stress treatment, three-week-old *Arabidopsis* seedlings grown on GM agar plates were transferred to 37°C under a photon flux density of 40±10 µmol photons m^−2^ s^−1^ [16], [17]. For the chemical treatments, 10-day-old seedlings were placed in a Petri dish filled with distilled water containing MG132 (200 µM; Merck Millipore, Darmstadt, Germany), epoxomicin (20 µM; Peptide Institute, Minoh, Japan), MG115 (200 µM; Peptide Institute), leupeptin (200 µM; Peptide Institute) or phenylmethylsulfonyl fluoride (PMSF; 200 µM, New England BioLabs, Ipswich, Massachusetts, United States) and subsequently incubated under illumination (50 µmol photons m^−2^ s^−1^). The control treatment consisted of 1% (v/v) dimethyl sulfoxide (DMSO; Wako, Osaka, Japan), which was used to dissolve the chemicals.

### Protein immunoblot analysis

Total protein was extracted from *Arabidopsis* seedlings and protoplasts in 1.5× Laemmli buffer containing 9 M urea. The extracts were centrifuged at 22,200×*g* for 30 min at 22°C and boiled at 95°C for 3 min. The resultant extracts, which corresponded to a fresh weight (FW) of 4 mg of seedling, were separated by sodium dodecyl sulfate-polyacrylamide gel electrophoresis (SDS-PAGE). Immunoblotting was performed using a polyclonal anti-DREB2A antibody (1∶1,500 dilution, see Methods S1 for a description of the antibody production) or a polyclonal anti-GFP antibody (1∶1,000 dilution, [21]) as the primary antibody and a goat anti-rabbit IgG peroxidase-conjugate (1∶10,000 dilution, Sigma-Aldrich, St. Louis, Missouri, United States) as the secondary antibody. The signals were developed using ECL Plus (GE Healthcare Life Sciences, Pittsburgh, Pennsylvania, United States) according to the manufacturer's instructions and detected using an image analyzer (LAS-3000, Fujifilm Life Science, Tokyo, Japan). Rehybridization was performed after stripping the antibodies with stripping buffer (100 mM 2-mercaptoethanol, 2% SDS, 62.5 mM Tris-HCl, pH 6.7) at 70°C for 30 min. Ponceau S (Sigma-Aldrich) staining of membranes was performed according to the manufacturer's instructions. The intensity of each band was measured with an image-processing and analysis software package (ImageJ, free software: http://rsb.info.nih.gov/ij/index.html). The ratio was calculated by normalizing the intensity of each DREB2A band to the intensity of the corresponding rbcL band.

### Total RNA extraction, RNA gel blot analysis and quantitative real-time reverse transcription PCR (qRT-PCR)

Total RNA isolation using RNAiso Plus (Takara Bio, Shiga, Japan), RNA gel blot analysis and qRT-PCR were conducted as previously described [18].

### Degradation kinetics

To evaluate the kinetics of DREB2A degradation, transgenic *Arabidopsis* suspension-cultured T87 cells expressing GFP were pretreated at 37°C for 2 h and subsequently incubated for 1 h in growth medium supplemented with 200 µg ml^−1^ cycloheximide (CHX; Sigma-Aldrich). The cells were then transferred to growth medium containing 100 µM MG132 or DMSO (solvent control) and CHX. After 1 h of treatment at 37°C, the cells were transferred to 22°C or maintained at 37°C and samples were collected at several time points.

### Transient expression in protoplasts and fluorescence microscopy observations

Transient expression assays using protoplasts derived from *Arabidopsis* mesophyll cells were performed according to the method described by Yoo et al. (2007) [22] with the modifications described previously [23]. Plasmids expressing GFP-DREB2A fusion proteins under the control of the CaMV *35S* promoter and the tobacco mosaic virus Ω sequence were constructed as described in Methods S1. A plasmid expressing Histone 2B fused to monomeric RFP (mRFP), which was cotransfected as a nuclear localization marker, was constructed as described in Methods S1. Fluorescence images of protoplasts expressing the GFP and mRFP fusion proteins were obtained using a confocal laser scanning microscope (LSM 510, Carl Zeiss, Jena, Germany, GFP fluorescence: 488 nm excitation filter and BP505–530 nm emission filter; RFP fluorescence: 543 nm excitation filter and BP560–615 nm emission filter).

## Results

### DREB2A protein accumulates in plants subjected to dehydration and heat stress

To analyze the levels of DREB2A in *Arabidopsis*, we developed a rabbit polyclonal antibody against a region of DREB2A spanning amino acid residues 166–335 that does not contain the well-conserved N-terminal domains or the NRD (Figure S1A). The activity and specificity of our antibody were verified by immunoblot analysis of the immunoprecipitated fractions from *35S:GFP* and *35S:GFP-DREB2A* transgenic plants [16] (Figure S1B). Additionally, the anti-DREB2A antibody had no significant cross-reactivity with DREB2B, which is the closest homolog of DREB2A [24] (Figure S1C).

**Figure 1:**
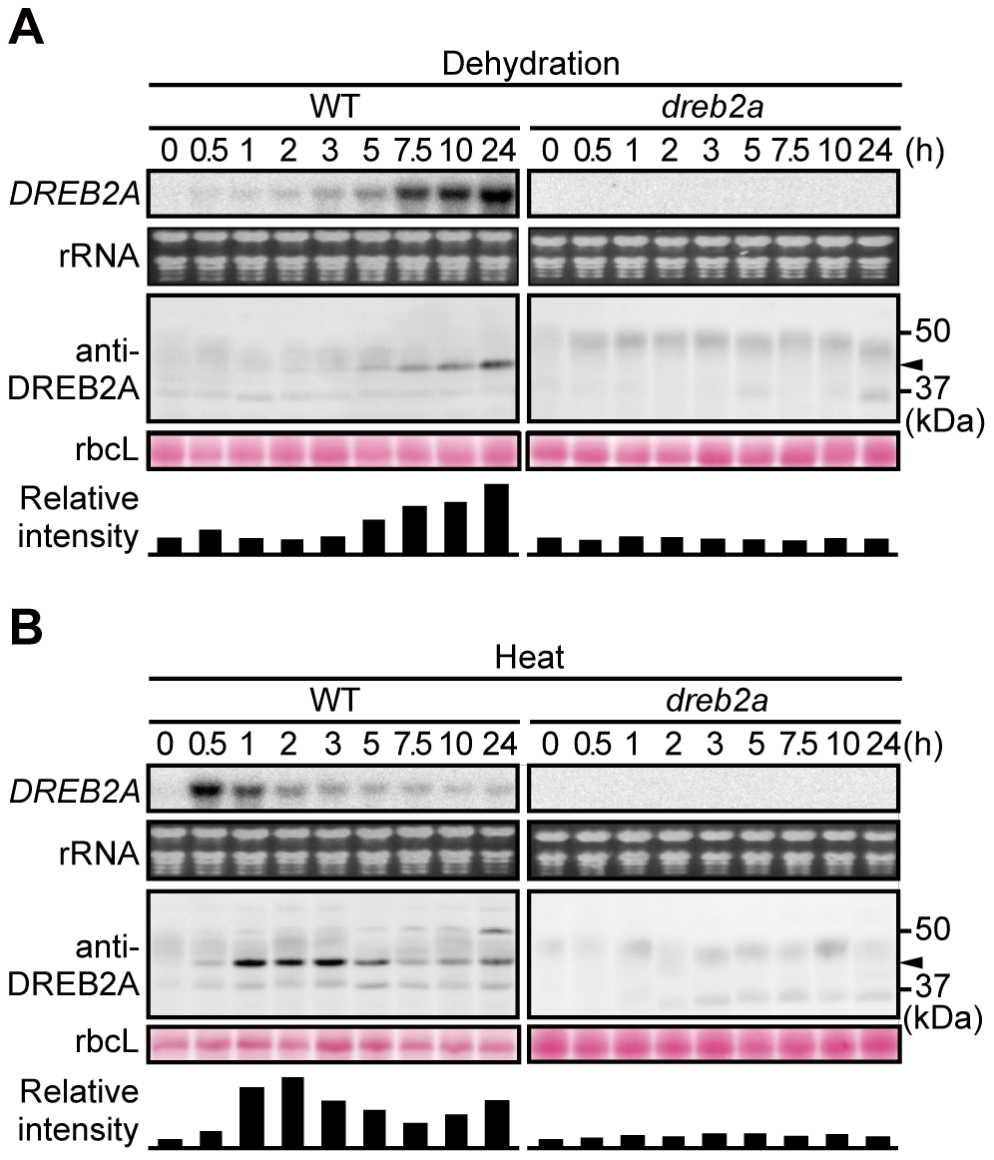
Accumulation of endogenous DREB2A under conditions of dehydration and heat stress. Three-week-old wild-type (WT) (left panel) and *dreb2-1* (right panel) seedlings were subjected to either dehydration (A) or heat stress (37°C) (B) for the indicated times. The upper part of each panel shows *DREB2A* mRNA levels. Each lane contains 10 µg of total RNA, and the rRNA bands (visualized by ethidium bromide (EtBr) staining) are shown as loading controls. The lower part of each panel shows the accumulation levels of DREB2A detected by immunoblot analysis using an anti-DREB2A antibody; the arrowhead indicates the major band of DREB2A. Each lane contains a total protein extract corresponding to 4 mg seedling fresh weight (FW). The Rubisco large subunit (rbcL) bands visualized by Ponceau S are shown as loading controls. Similar results were obtained in three independent experiments. The bars below the bands show the intensity of the DREB2A band relative to the corresponding rbcL band in each lane.

Next, we examined the accumulation pattern of DREB2A in WT *Arabidopsis* seedlings subjected to dehydration (Figure 1A) or heat stress (Figure 1B). Different accumulation patterns of DREB2A were observed for the two stress treatments: DREB2A accumulated gradually over the course of the dehydration stress while plants subjected to heat stress experienced a rapid and intense accumulation of DREB2A within 1 to 2 h followed by a gradual reduction in DREB2A protein levels. No corresponding bands were detected in the *dreb2a-1* mutant [17] (Figure 1). The protein accumulation patterns of DREB2A were similar to those of its transcript levels for both stress treatments; however, there were time lags for the protein and transcript level changes (Figure 1). Because overexpressed DREB2A does not accumulate under non-stressful conditions [16], the observed accumulation of DREB2A suggests that this protein was stabilized by the heat and dehydration stresses.

### DREB2A protein degradation is inhibited at high temperatures

**Figure 2:**
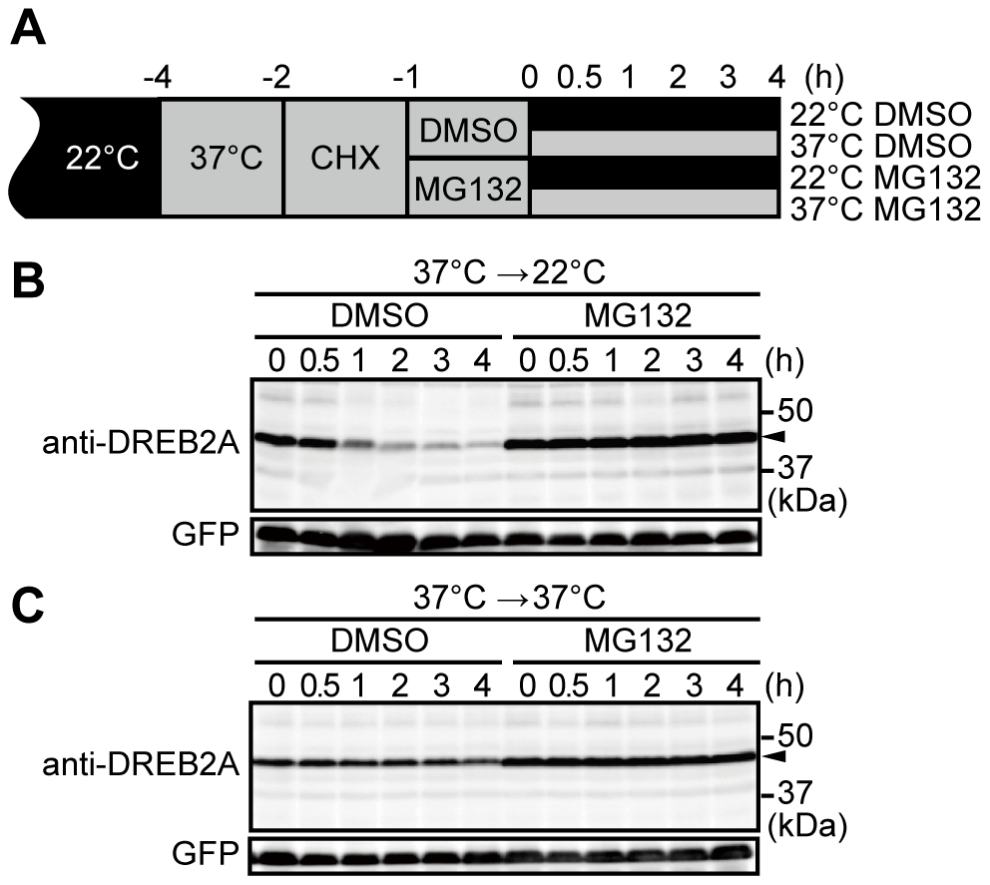
DREB2A stability is increased under heat stress. (A) Schematic representation of the treatments used in the degradation assay. (B) Degradation of DREB2A in T87 cells constitutively expressing GFP after a shift from high temperatures (37°C) to normal conditions (22°C). The levels of DREB2A and GFP (loading control) accumulation were determined by immunoblot analyses with specific antibodies. The arrowhead indicates the major band of DREB2A. (C) Degradation of DREB2A in T87 cells that were kept at high temperatures (37°C).

To confirm the increased stability of DREB2A under stressful conditions, we compared the degradation kinetics of DREB2A in plants subjected to heat stress and normal conditions. To ensure rapid responses to the treatments, we used *Arabidopsis* T87 suspension-cultured cells for the analysis. T87 cells were pretreated at 37°C for 1 h to facilitate the accumulation of DREB2A protein (Figure 2A). The protein synthesis inhibitor cycloheximide (CHX) was then added, and, after 1 h, the cells were further treated with the proteasome inhibitor MG132 or solvent (DMSO) for 1 h. Following these treatments, the cells were maintained at 37°C or shifted to 22°C and DREB2A protein levels were measured. In the absence of MG132, DREB2A levels decreased slightly at 37°C but decreased rapidly at 22°C (Figure 2B, C). In contrast, MG132 almost completely inhibited the degradation of DREB2A under both conditions (Figure 2B, C). These results confirm that the stability of DREB2A is markedly increased at high temperatures.

### Proteasome-dependent degradation of DREB2A is regulated in the nucleus

**Figure 3:**
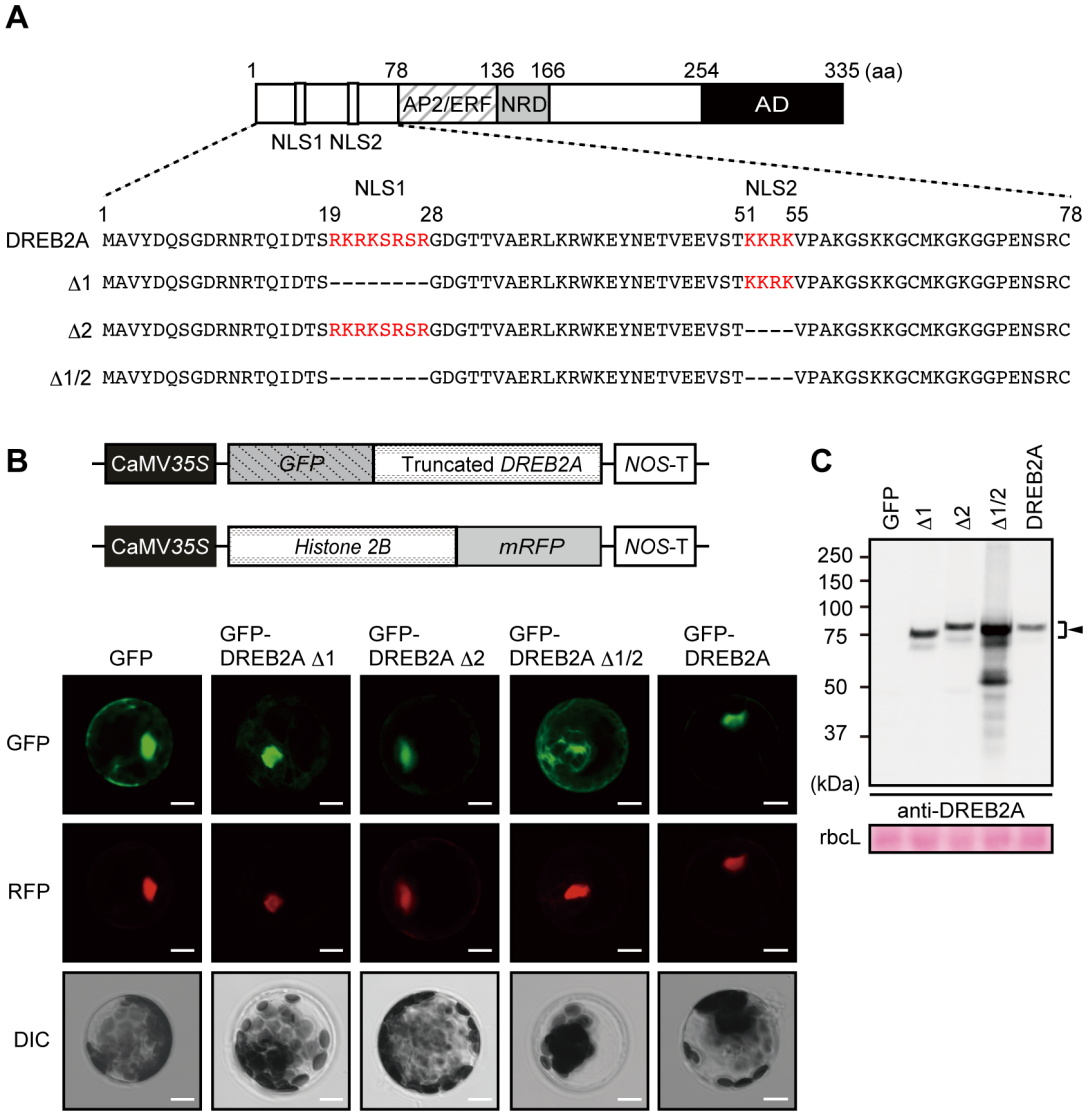
Nuclear localization of DREB2A is redundantly regulated by two nuclear localization signals (NLSs) and associated with a low DREB2A protein level. (A) Schematic representation of the DREB2A protein showing the amino-terminal region, which contains two putative NLSs. The NLSs predicted by Liu et al. (1998) are shown in red. (B) Localization of GFP-DREB2A fusion proteins transiently expressed in *Arabidopsis* mesophyll protoplasts. A plasmid that constitutively expresses Histone 2B fused to mRFP was cotransfected into the protoplasts as a positive control for transfection and nuclear localization. Confocal images of the GFP field (GFP) and the RFP field (RFP) and differential interference contrast (DIC) images are shown from top to bottom. Bars = 20 µm. (C) Differences in the amounts of GFP-fused DREB2A proteins transiently expressed in *Arabidopsis* mesophyll protoplasts. Accumulation levels of the DREB2A proteins were determined by immunoblot analysis using the anti-DREB2A antibody. A plasmid that expresses the *Luciferase* reporter gene under the control of the CaMV *35S* promoter was co-transfected as an internal control and the Luciferase activity was used for protein loading adjustment. The arrowhead indicates the major band of the GFP-fused DREB2A proteins. The Rubisco large subunit (rbcL) bands were visualized by Ponceau S and are shown as loading controls.

We previously reported that the E3 ubiquitin ligases DRIP1 and DRIP2 target DREB2A to 26S proteasome-mediated proteolysis under normal conditions [18]. DRIP1 and DRIP2 are predominantly located in the nucleus in *Arabidopsis* plants and physically interact with DREB2A in onion cell nuclei under normal conditions [18]. However, it is not clear whether this is the only system responsible for DREB2A degradation: it is possible that other E3s may be involved or that the degradation of nascent DREB2A occurs in the cytosol before its nuclear import. We first tested the necessity of nuclear import for DREB2A degradation. DREB2A has two putative nuclear localization signals (NLSs), NLS1 and NLS2, in the N-terminal region [25] (Figure 3A). To confirm the function of these two NLSs, we constructed a series of plasmids expressing GFP-DREB2A fusion proteins lacking either one or both of the NLSs under the control of the CaMV *35S* promoter (Figure 3A, B). We then observed the localization patterns of these proteins in *Arabidopsis* mesophyll protoplasts using a transient expression system (Figure 3B). We used a Histone 2B-mRFP fusion protein as a nuclear localization control [23] (Figure 3B). The fluorescence of GFP-DREB2A Δ1 and GFP-DREB2A Δ2, which both lack one NLS, was colocalized with that of mRFP in the nucleus; similar colocalizations were observed for wild-type GFP-DREB2A. In contrast, the fluorescence of a GFP-DREB2A fusion protein that lacks both of the NLSs (GFP-DREB2A Δ1/2) was detected predominantly in the cytosol. These results suggest that either of the NLSs is sufficient to target DREB2A to the nucleus.

Next, we examined the accumulation levels of these GFP-DREB2A fusion proteins (Figure 3C). The accumulation levels of wild-type GFP-DREB2A, GFP-DREB2A Δ1 and GFP-DREB2AΔ2 were very similar; however, GFP-DREB2A Δ1/2 accumulated to considerably higher levels (Figure 3C). We also verified the activity of these GFP-fused DREB2As using transient reporter assays in *Arabidopsis* mesophyll protoplasts (Figure S2). GFP-DREB2A transactivated the 3×DRE-GUS reporter gene [25] and GFP-DREB2A CA exhibited twice as much activity as GFP-DREB2A. This result is consistent with a previously obtained result for untagged DREB2A [16], suggesting that GFP-fused DREB2A proteins retain the character of untagged DREB2As. GFP-DREB2A Δ1 and GFP-DREB2A Δ2 had slightly higher but similar transactivation activity as wild-type GFP-DREB2A. In contrast, the transactivation activity of GFP-DREB2A Δ1/2 was almost as low as that of the vector control. Thus, losing either of the two NLSs does not substantially impact transactivation and the activity of these GFP-DREB2A mutants is correlated with their localization. These results indicate that mutant DREB2A lacking either NLS is still trafficked to the nucleus and controlled by 26S proteasome-mediated proteolysis. In addition, the stability of GFP-DREB2A Δ1/2 implies that the DREB2A protein may be more stable in the cytosol than in the nucleus.

**Figure 4:**
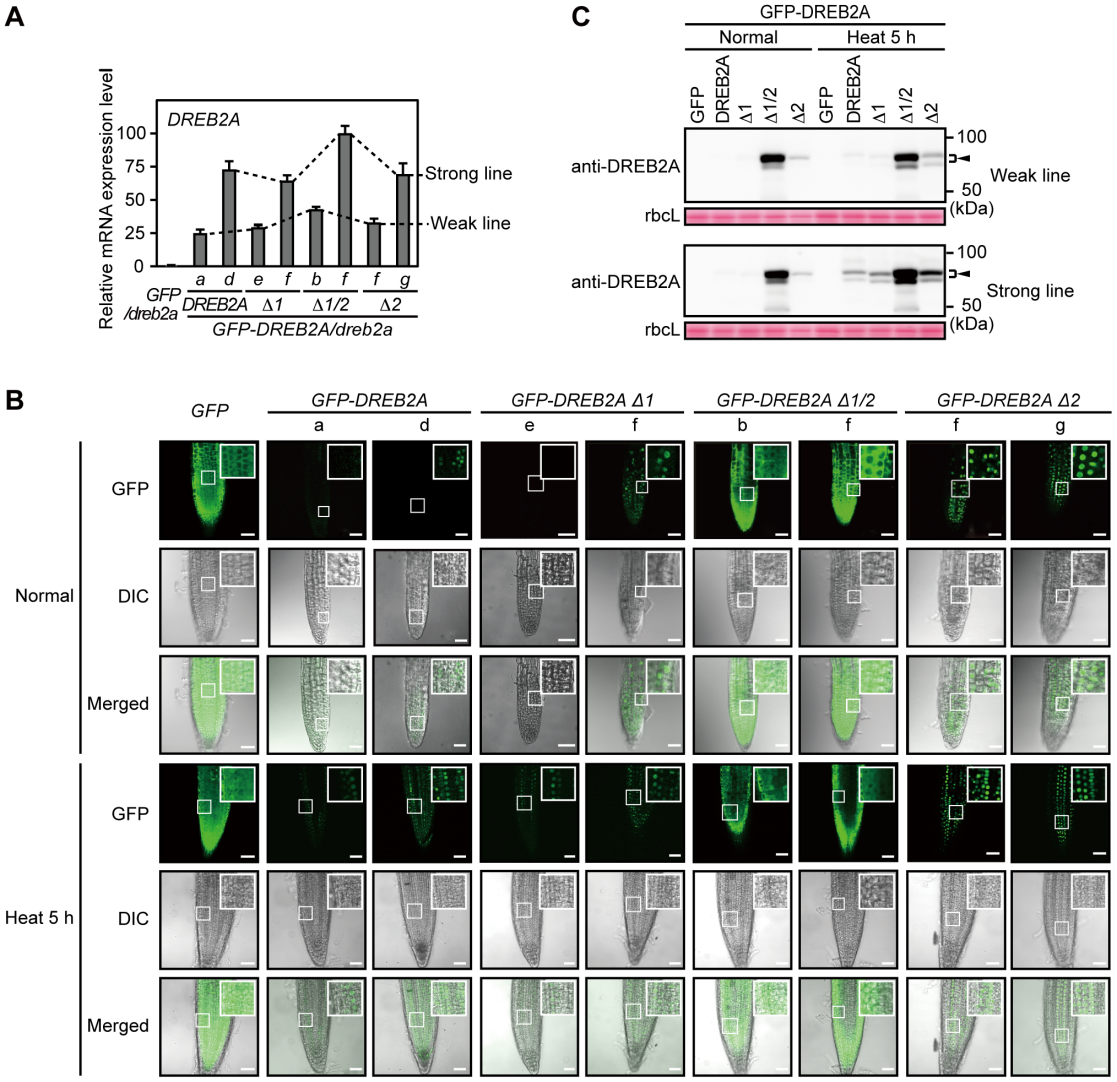
Relationship between subcellular localization and accumulation levels of the DREB2A protein in plants. Wild-type and deletion mutants of DREB2A were fused to GFP and constitutively expressed in the *dreb2a-1* background and their subcellular localization and accumulation levels were analyzed. (A) The expression levels of the transgenes were determined using quantitative RT-PCR. The amounts of the transcripts were normalized to those of *ACT8*. The values are presented as the means of triplicate technical repeats and the error bars indicate standard deviations (SDs). The highest expression level was set to 100. Similar results were obtained in two independent experiments. Lowercase letters indicate two independent transgenic lines that express the transgenes strongly (Strong line) and weakly (Weak line). (B) Localization of the GFP-DREB2A fusion proteins in transgenic *Arabidopsis* plants subjected to normal conditions and heat stress. Confocal images of the GFP field (GFP), differential interference contrast (DIC) images and merged images are shown from top to bottom. Bars = 50 µm. (C) Amount of the DREB2A proteins in transgenic *Arabidopsis* plants subjected to normal conditions and heat stress. Accumulation levels of the DREB2A proteins were determined by immunoblot analysis using the anti-DREB2A antibody. The arrowhead indicates the major band of the GFP-fused DREB2A proteins in the transgenic *Arabidopsis* plants. Rubisco large subunit (rbcL) bands visualized by Ponceau S are shown as loading controls.

Next, we examined the effect of these deletions on the subcellular localization and stability of DREB2A using transgenic *Arabidopsis* lines constitutively expressing the GFP-DREB2As in the *dreb2a-1* background (*GFP-DREB2As/dreb2a*) (Figure 4A). We selected two independent lines that either weakly or strongly expressed each transgene. Then, we observed the localization of the GFP-DREB2As in these plants under normal conditions (Figure 4B). Weak fluorescence was observed in the nucleus for wild-type GFP-DREB2A, GFP-DREB2A Δ1 and GFP-DREB2A Δ2, whereas strong fluorescence was observed in the cytosol for GFP-DREB2A Δ1/2 (Figure 4B). These results were consistent with the localization pattern observed in the protoplast transient expression system (Figure 3B). Under conditions of heat stress, strong fluorescence was observed in the nucleus for wild-type GFP-DREB2A, GFP-DREB2A Δ1 and GFP-DREB2A Δ2, whereas consistent fluorescence was observed in the cytosol for GFP-DREB2A Δ1/2 (Figure 4B). We then analyzed the accumulation of the GFP-DREB2A fusion proteins under normal conditions and conditions of heat stress (Figure 4C). Consistent with the results of transient expression in protoplasts (Figure 3C), levels of GFP-DREB2A, GFP-DREB2A Δ1 and GFP-DREB2A Δ2 were significantly lower than levels of GFP-DREB2A Δ1/2 under normal conditions. Under conditions of heat stress, the levels of GFP-DREB2A, GFP-DREB2A Δ1 and GFP-DREB2A Δ2 increased while those of GFP-DREB2A Δ1/2 were not significantly altered. However, GFP-DREB2A Δ1/2 protein levels were much higher than those of the other fusion proteins. These results suggest that all of the GFP-DREB2As (except GFP-DREB2A Δ1/2) that can be imported to the nucleus are still partially degraded under conditions of heat stress. These results indicate that the regulation of DREB2A stability requires nuclear import irrespective of the presence of heat stress.

### The E3 ligases DRIP1 and DRIP2 are involved in the degradation of DREB2A under conditions of stress and normal conditions

**Figure 5:**
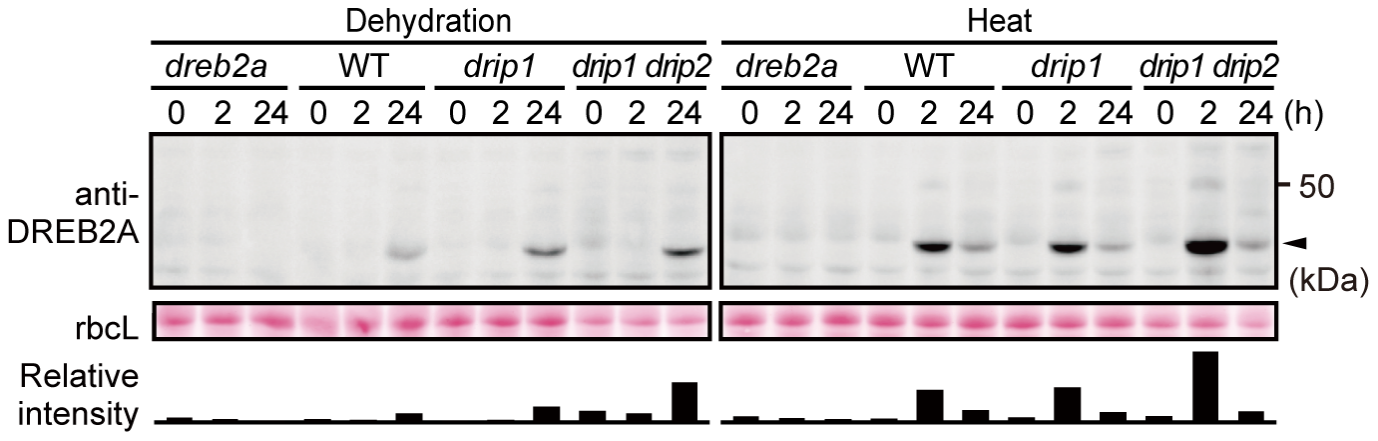
Accumulation levels of DREB2A protein in *drip1* and *drip1 drip2* mutants subjected to dehydration and heat stress. Three-week-old WT, *dreb2a-1*, *drip1* and *drip1 drip2* seedlings were subjected to either dehydration (left panel) or heat stress (right panel). Accumulation levels of the DREB2A protein were determined by immunoblot analysis using the anti-DREB2A antibody. The arrowhead indicates the major band of DREB2A; rbcL bands visualized by Ponceau S are shown as loading controls. The bars below the bands show the intensity of the DREB2A band relative to the corresponding rbcL band in each lane.

Although DRIP1 and DRIP2 mediate DREB2A degradation under normal conditions [18], their role in the turnover of DREB2A under stressful conditions and normal conditions is unknown. Thus, we examined changes in DREB2A accumulation in *drip1* single and *drip1 drip2* double mutants subjected to dehydration and heat stress (Figure 5). Under both conditions, the peak levels of DREB2A were higher in *drip1* and *drip1 drip2* mutants and corresponded to the number of disrupted *DRIP* genes (Figure 5); this indicates that DRIP1 and DRIP2 are involved in the turnover of DREB2A, even under conditions of stress. Moreover, we found that under conditions of heat stress, DREB2A levels are lower at 24 h than at 2 h, even in the *drip1 drip2* double mutant. To examine whether this reduction in DREB2A protein levels during the late phase of heat stress depends on 26S proteasome-mediated proteolysis, we treated WT *Arabidopsis* plants subjected to heat stress with the proteasome inhibitor MG132 (Figure S3). We found that the reduction in DREB2A accumulation was inhibited by MG132. These results indicate that the 26S proteasome-mediated degradation of DREB2A still occurs in the nucleus under conditions of stress and that unknown E3 ligases other than DRIP1 and DRIP2 may be involved in this process during the late phase of heat stress.

### DREB2A protein accumulation affects the strength of downstream gene induction in response to dehydration and heat stress

**Figure 6:**
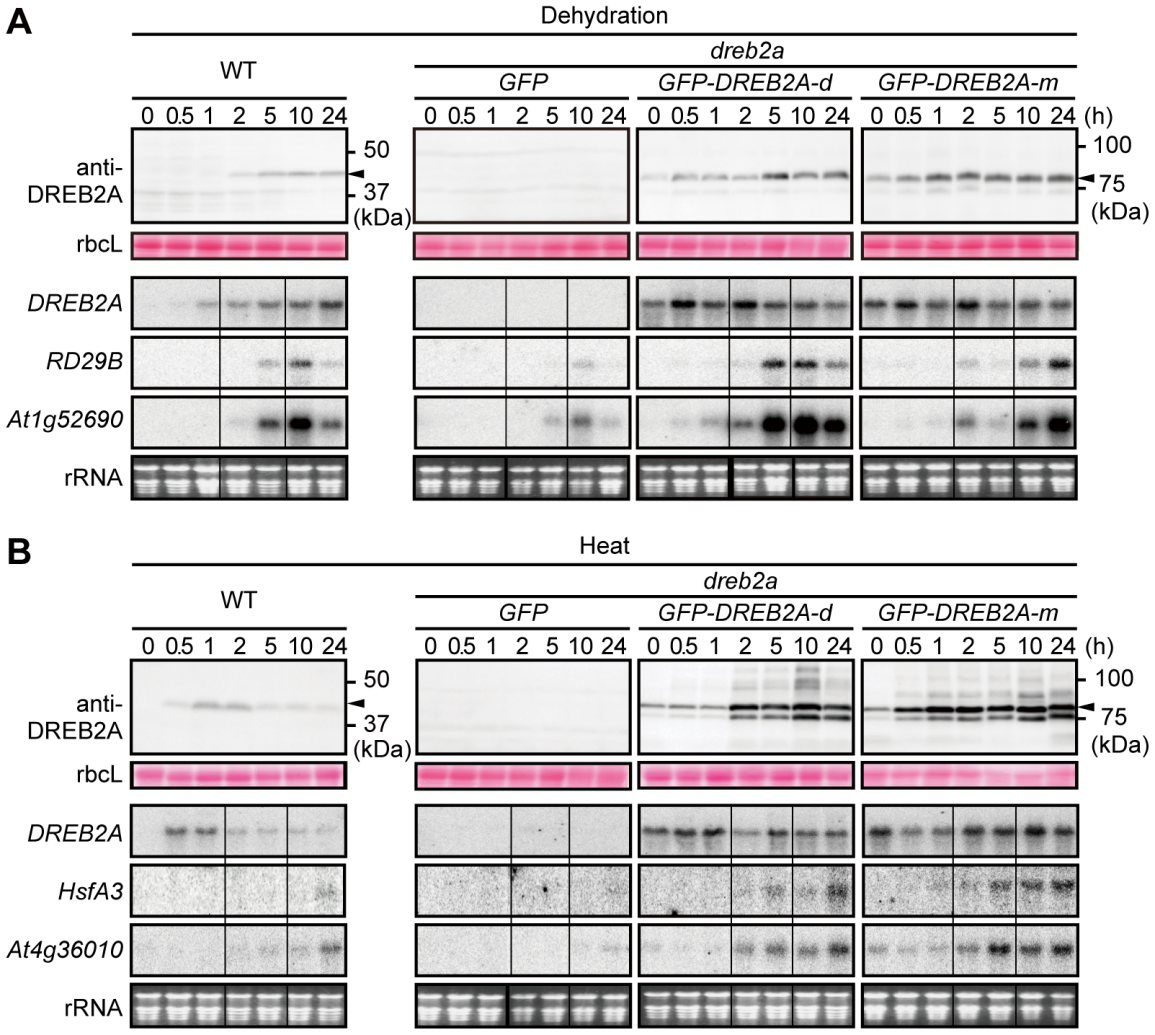
Overaccumulation of GFP-DREB2A enhances the induction of target gene expression in plants subjected to dehydration and heat stress. Three-week-old seedlings of WT, *GFP/dreb2a-1* and two independent lines of *GFP-DREB2A/dreb2a* were subjected to either dehydration (A) or heat stress (37°C) (B) for the indicated times. The upper part of each panel shows the accumulation of DREB2A or GFP-DREB2A. The arrowhead indicates the major band of either endogenous DREB2A in WT or GFP-DREB2A in *GFP-DREB2A/dreb2a*. Each lane contains a total protein extract corresponding to 4 mg seedling FW. rbcL bands visualized by Ponceau S are shown as loading controls. The lower part of each panel shows the mRNA levels of *DREB2A* or the following target genes: dehydration-responsive *RD29B* and *At1g52690* (A) or heat-responsive *HsfA3* and *At4g36010* (B). Each lane contains 10 µg of total RNA and the rRNA bands (visualized by EtBr staining) are shown as loading controls. Similar results were obtained in three independent experiments.

Next, we examined the effect of DREB2A protein stabilization on the induction of target genes in *Arabidopsis* plants. Because endogenous *DREB2A* itself is inducible by stress, we used transgenic *Arabidopsis* plants that constitutively express *GFP-DREB2A* in the *dreb2a* mutant background (*i.e.*, *GFP-DREB2A*/*dreb2a*) to abstract the effect of stabilization. First, we compared the accumulation patterns of DREB2A under conditions of dehydration and heat stress in WT plants and two independent *Arabidopsis GFP-DREB2A/dreb2a* lines (d and m) that strongly expressed the transgene (Figure 6). A gradual accumulation of GFP-DREB2A was observed in plants subjected to dehydration, and a marked accumulation of GFP-DREB2A was observed in heat-stressed plants (Figure 6). Taken together with Figure 4B and C, these observations indicate that DREB2A stability increases in response to heat stress and dehydration. GFP-DREB2A accumulation began earlier in these lines than in WT plants subjected to dehydration (Figure 6A). During heat stress, GFP-DREB2A levels remained high for 24 h in the *GFP-DREB2A/dreb2a* lines; in contrast, DREB2A levels in the WT plants decreased after 5 h (Figure 6B). Although GFP-DREB2A may be more stable than DREB2A, the accumulation pattern of DREB2A in WT plants under these conditions appears to be determined by a combination of transcriptional and post-transcriptional effects (Figures 1 and 6).

To determine whether the level of DREB2A accumulation affects the induction of downstream genes, we selected four DREB2A target genes that contain the DRE sequence in their promoter region and are highly upregulated in *35S:DREB2A CA* plants under normal conditions and markedly downregulated in *dreb2a* mutants subjected to dehydration or heat stress. Two of these targets, *RD29B* and *At1g52690*, are dehydration inducible; the other two, *HsfA3* and *At4g36010*, are heat inducible [16], [17] (Figure 6). During dehydration stress, the *RD29B* and *At1g52690* genes were induced earlier and expressed more strongly in the transgenic plants than in the WT plants. Although the expression patterns of these target genes differed between the two transgenic lines in the time course experiment, this difference was not always observed in triplicate experiments and was therefore not considered significant. During heat stress, the *HsfA3* and *At4g36010* genes were also induced earlier and expressed more strongly in the transgenic plants than in the WT plants. These results were consistent with the higher accumulation of DREB2A in the transgenic plants than in the WT plants (Figure 6). This positive relationship between the amount of DREB2A protein and the expression levels of the target genes suggests that the accumulation of DREB2A in response to stress is an important factor in the regulation of target gene expression.

### DREB2A protein accumulation induced by treatment with proteasome inhibitors is not sufficient to induce the expression of target genes under normal conditions

**Figure 7:**
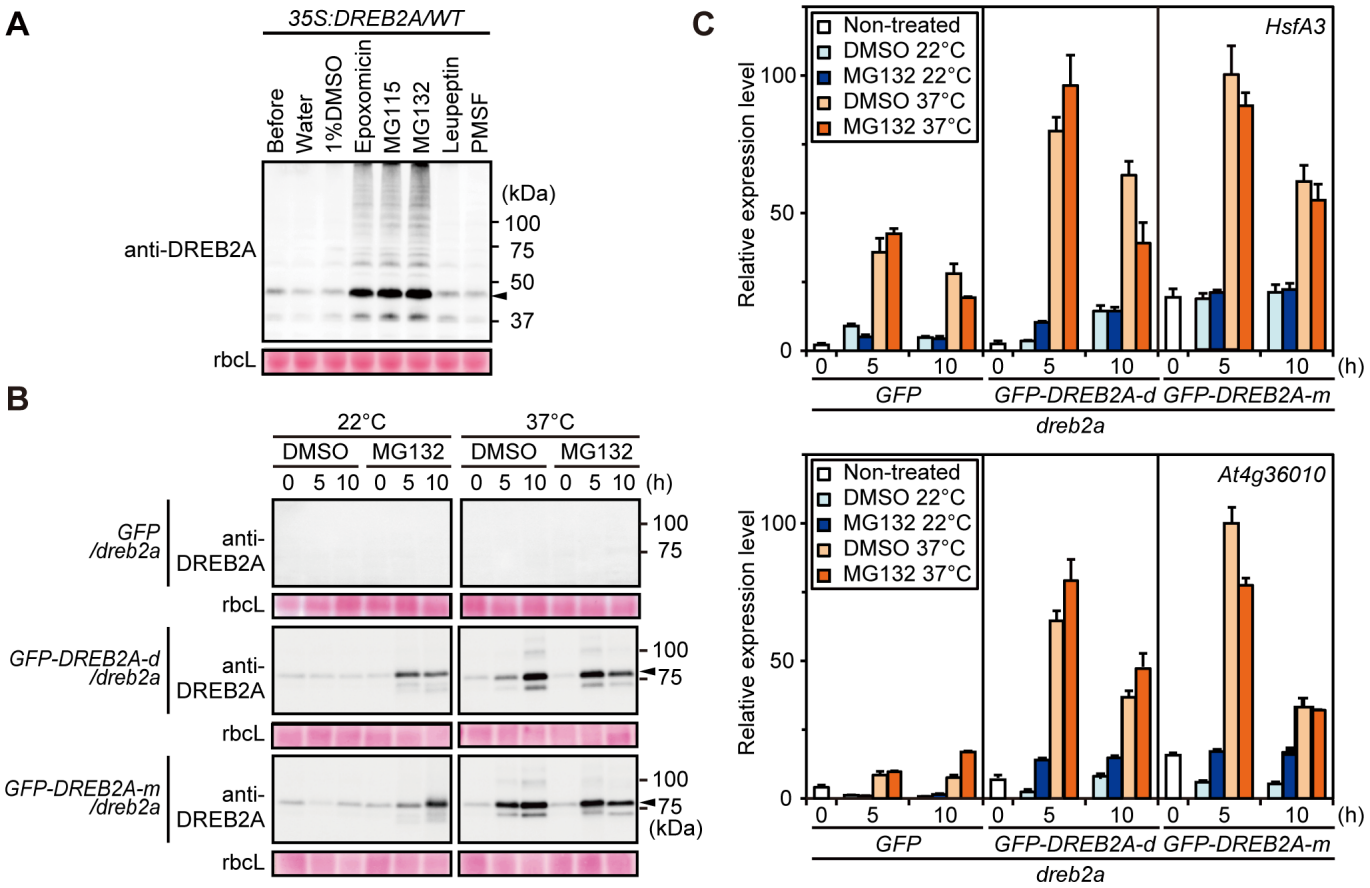
MG132-induced accumulation of GFP-DREB2A is not sufficient for the induction of DREB2A target genes under normal conditions. (A) The effects of various protease and proteasome inhibitors on the degradation of the DREB2A protein in transgenic *Arabidopsis* seedlings. Total protein was extracted from 10-day-old DREB2A or WT seedlings treated with water, 1% (v/v) DMSO, proteasome inhibitors (20 µM epoxomicin, 200 µM MG115 or 200 µM MG132) or protease inhibitors (200 µM leupeptin or 200 µM PMSF) under normal growth conditions for 10 h. A protein extract was prepared from non-treated seedlings (Before) to serve as a control. The protein extracts corresponded to 4 mg seedling FW and were loaded onto an SDS-PAGE gel. Immunoblot analysis was performed using the anti-DREB2A antibody. The arrowhead indicates the major band of DREB2A. rbcL bands visualized by Ponceau S are shown as loading controls. (B) Accumulation levels of GFP-DREB2A. Ten-day-old seedlings of *GFP/dreb2a* and two independent lines of *GFP-DREB2A/dreb2a* were treated with or without 200 µM MG132 under normal (22°C) conditions or conditions of heat stress (37°C). The arrowhead indicates the major band of GFP-DREB2A. (C) The effects of MG132 treatment on the expression of DREB2A target genes. The mRNA levels of two heat-inducible DREB2A target genes were determined using quantitative RT-PCR. The amounts of the transcripts were normalized to those of *ACT8*. The values represent the means of triplicate technical repeats and the error bars indicate SDs. The highest expression level was set to 100 for each gene. Similar results were obtained in two independent experiments.

We analyzed whether DREB2A accumulation caused by proteasome inhibitors is sufficient for the induction of target genes under normal conditions. However, because proteasome inhibitors such as MG132 partially inhibit several proteases in addition to the 20S proteasome [26], we first tested the effects of different proteasome inhibitors (MG132, MG115 and epoxomicin) on DREB2A degradation. Leupeptin and PMSF, which are general inhibitors of serine and cysteine proteases, were also tested. Treatment with the general protease inhibitors did not lead to the accumulation of DREB2A protein; however, DREB2A accumulated to high levels after treatment with the proteasome inhibitors (Figure 7A). Therefore, we used two of these proteasome inhibitors (MG115 and MG132) for the following experiments to ensure that the consequences of the inhibitor treatments were specifically related to the inhibition of 26S proteasome-mediated degradation. As shown in Figures 7B and S4A, the treatment of *35S:GFP-DREB2A* transgenic plants with proteasome inhibitors resulted in similar levels of DREB2A accumulation under normal conditions and conditions of heat stress. The expression of target genes was minimally induced by proteasome inhibitor treatment under normal conditions; in contrast, target genes were induced to a greater extent under conditions of heat stress (Figures 7C, S4B). Furthermore, to exclude the possibility that treatment with the proteasome inhibitor blocked the activity of general transcriptional machinery, we treated the plants with the proteasome inhibitors during heat stress. The expression of target genes under heat stress was induced to similar levels with and without proteasome inhibitors (Figures 7C, S4B). These results demonstrate that DREB2A accumulation alone is not sufficient for the substantial induction of its target genes and suggest that another activation process is required.

## Discussion

Although the post-translational regulation of *DREB2A* is important for its activation, and the stabilization of DREB2A is involved in its activation [16]–[18], little information is available on the accumulation of DREB2A in plants subjected to dehydration and heat stress. Using a newly developed anti-DREB2A antibody, we were able to detect the accumulation of endogenous DREB2A *in planta* (Figure 1). In this experiment, under conditions of dehydration and heat stress, the accumulation patterns of the DREB2A protein were similar to those of its transcript, albeit with a time lag. This result indicates that the accumulation of DREB2A is dependent on its transcript levels. However, we also obtained clear evidence of the stress-dependent stabilization of the DREB2A protein. The incubation of T87 cells at 37°C resulted in an accumulation of DREB2A protein that decreased rapidly at 22°C but remained stable at 37°C (Figure 2). We also found that the DREB2A levels were low under normal conditions but increased under conditions of dehydration and heat stress in the *GFP-DREB2A/dreb2a* transgenic lines that constitutively overexpress *DREB2A* (Figure 6). These results clearly indicate that the stress signal is important for the stabilization of DREB2A proteins.

In contrast, protein levels decreased in WT plants after 5 h of heat treatment (Figure 1B) and MG132 blocked the gradual decrease in DREB2A protein levels at 37°C (Figures 2C, S3). An analysis of DREB2A protein levels in the *drip1* and *drip1 drip2* mutants showed that DRIP1 and DRIP2 are involved in the degradation of DREB2A under conditions of dehydration and heat stress (Figure 5). However, after 24 h of heat treatment, DREB2A levels in the *drip1 drip2* mutant decreased to levels similar to those observed in the WT plants (Figure 5), which suggests that factors other than DRIP1 and DRIP2 are involved in mediating DREB2A degradation (at least under conditions of heat stress).

DREB2A proteins lacking NLSs provided additional information on DREB2A degradation. The GFP-DREB2A Δ1/2 protein experienced impaired nuclear localization and increased stabilization under normal and stressful conditions (Figures 3, 4). GFP-DREB2A Δ1/2 accumulated to high levels, and its accumulation was only mildly affected by heat stress (Figure 4C); furthermore, its accumulation appeared to exceed that of the wild-type DREB2A protein subjected to stressful conditions (Figure 4C). Conversely, GFP fusions with wild-type and other variants of DREB2A were exclusively localized to the nucleus (irrespective of the treatment conditions) and accumulated to significantly higher levels after heat stress treatment (Figure 4B). Although the DREB2A protein is synthesized in the cytosol, it can be imported to the nucleus and degraded regardless of the presence or absence of stress signals. DRIP1 was shown to localize to the nucleus [18], and other factors required for DREB2A degradation may also localize to the nucleus. These data also support the idea that the accumulation of DREB2A in the nucleus is limited, even under stressful conditions.

Previously, it was unclear whether the stabilization of DREB2A is sufficient for the induction of its target genes or whether an additional activation mechanism is required. Our results suggest that subsequent activation steps may be required in addition to the complex regulation of DREB2A stability. The accumulation of DREB2A in the *GFP-DREB2A/dreb2a* plants subjected to stress enhanced the expression of target genes, suggesting that the amount of DREB2A influences the strength of target gene expression (Figure 6). On the other hand, we also showed that the accumulation of stabilized DREB2A in plants treated with proteasome inhibitors minimally induced the expression of target genes under normal conditions (Figures 7, S4). Additionally, we showed that a significant amount of the DREB2A protein accumulates when WT plants are subjected to dehydration and heat stress (Figure 1). However, different target genes of DREB2A are activated under conditions of dehydration and heat, while DREB2A CA can induce the expression of all target genes even under non-stressful conditions [17]. These findings suggest that an additional regulatory step is involved in the stress-responsive expression of DREB2A target genes. Thus, we propose that the post-translational regulation of DREB2A involves separate stabilization and activation steps, both of which are important for target gene expression in response to stress signals in plants.

An example of a transcription factor that requires activation in addition to stabilization is ABA-insensitive5 (ABI5), a protein that positively regulates ABA signaling. Under normal conditions, the ABI5 protein is subjected to proteasomal degradation mediated by the E3 ubiquitin ligases KEEP ON GOING (KEG) and DWD hypersensitive to ABA (DWA). ABA promotes ABI5 accumulation by promoting KEG degradation and inducing *ABI5* transcription [27]. ABA also induces the phosphorylation of ABI5, enabling a critical regulatory step in its activation. Multiple levels of regulatory mechanisms controlling the stability and activity of proteins have also been found in mammalian cells. One example of these is the sterol regulatory element-binding protein (SREBP) family, which consists of transcription factors that regulate the expression of many fatty acid and triglyceride synthesis-related genes. The stability of SREBPs is known to be regulated by competition between *N*-acetylation and ubiquitination; in contrast, the activity of SREBPs is regulated both positively and negatively by other modifications [28]–[30]. Thus, it is possible that protein modifications are also involved in the regulation of DREB2A activity. Although no modifications of DREB2A in *Arabidopsis* have been reported to date, we observed additional DREB2A bands in the immunoblot analyses that may be indicative of unknown modifications of this protein (Figures 1, 6 and 7). In fact, a DREB2A homolog in *Pennisetum glaucum* have been reported to be phosphorylated *in vitro* and its phosphorylation reduces its DNA-binding activity [31]. The identification of DREB2A protein modifications and their effects may shed light on the molecular mechanisms of DREB2A stabilization and activation. Furthermore, the interaction of DREB2A with other proteins appears to contribute to its complex regulation. DRIP1/2, RADICAL-INDUCED CELL DEATH 1 (RCD1) and Med25 have been identified as DREB2A-interacting proteins [32], [33]. RCD1 interacts with the activation domain of DREB2A and Med25 is a component of the mediator complex involved in the recruitment of RNA polymerase II. Although the roles of these proteins in the activation of DREB2A are unclear, identifying the protein interaction network surrounding DREB2A will shed light on the role played by DREB2A in the regulation of stress responses and growth regulation in response to environmental fluctuations.

DREB2A is a member of the DREB subfamily in the ERF/AP2 family, which share a well-conserved DNA-binding domain [34]. DREB2 and DREB1/CBF are two major types of DREBs, however, in contrast to DREB2s, DREB1s/CBFs function in cold stress responses in *Arabidopsis*. They are highly induced by low temperatures and their ectopic expression results in the induction of target genes under non-stressful conditions; thus DREB1s/CBFs are considered to be mainly regulated at the transcriptional level. Therefore, the post-translational regulation of the stability and activity of DREB1s/CBFs in response to stress are not expected to have a significant impact on the expression of genes regulated by these proteins. On the other hand, DREB2A has two close homologs in *Arabidopsis*, DREB2B and DREB2C. These proteins share several conserved domains in addition to the DNA-binding domain, including the NRD-like domain, the sequences surrounding the two NLSs (CMIV-1 and CMIV-2) and CMIV-3 [34]. CMIV-3 is involved in the interaction of DREB2A with RCD1 [33]. In addition, other DREB2A homologs have been identified in various plants [35] and we recently showed that a DREB2A homolog from soybean has an NRD-like domain and undergoes post-translational regulation similar to that experienced by DREB2A [36]. Therefore, the results of the present study are likely applicable to other DREB2A homologs.

In this report, we demonstrated that DREB2A, which is degraded by 26S proteasome-mediated proteolysis under normal conditions, is stabilized under conditions of dehydration and heat stress. Although the amount of DREB2A protein is an important factor related to the expression level of its target genes, the stabilization of the protein is not sufficient for DREB2A activation. Thus, an as yet unknown activation mechanism, which may involve protein modifications or interactions with co-factors, is likely required in addition to stabilization (Figure S5). We expect that knowledge of such activation steps could provide insight into how DREB2A alternately regulates two distinct stress signaling pathways in response to dehydration and heat stress. As a stress-inducible transcription factor, DREB2A plays a critical role in the response of the *Arabidopsis* transcriptome to environmental stresses; however, the products of these target genes are detrimental to plant growth under non-stressful conditions [16]. The rapid turnover of DREB2A may be required to minimize the negative effects of DREB2A on plant growth while ensuring rapid responses to changing environmental conditions. Thus, further elucidation of the complex processes affecting transcriptional regulation by DREB2A will be critical for understanding the events that occur in response to multiple environmental stresses.

## Supporting Information

Figure S1 Specificity of the anti-DREB2A antibody. (A) Schematic representation of the DREB2A protein showing the region corresponding to amino acid residues 166–335 that was used to prepare the DREB2A polyclonal antibody (indicated by the double-headed arrow). NLS, nuclear localization signal; AP2/ERF, AP2/ERF DNA-binding domain; NRD, negative regulatory domain; AD, activation domain. (B) Evaluation of anti-DREB2A antibody activity. GFP or GFP-DREB2A was transiently expressed under the control of CaMV *35S* promoter in *Nicotiana benthamiana* leaves and immunoprecipitated using anti-GFP microbeads. The precipitated fraction was analyzed by immunoblot with the anti-DREB2A antibody (left panel). The result of subsequent stripping and re-hybridization with anti-GFP antibody is shown in the right panel. (C) Confirmation of the specificity of the DREB2A antibody. GFP, GFP-DREB2A or GFP-DREB2B was transiently expressed under the control of the CaMV *35S* promoter in *N. benthamiana* leaves and immunoprecipitated using anti-GFP microbeads. The immunoprecipitated fractions corresponding to 0.2 mg (GFP) or 2 mg (GFP-DREB2A and GFP-DREB2B) of leaves (FW) were loaded onto SDS-PAGE gels and analyzed by immunoblot with either the GFP (left panel) or DREB2A antibody.
(TIF)

Figure S2 Transcriptional activity was retained in the GFP-DREB2A protein lacking one of the two nuclear localization signals (NLSs), but not in the protein lacking both of the NLSs. The transactivation activity of the GFP fusion proteins of DREB2A lacking one or both of the NLSs was compared with that of wild-type DREB2A and DREB2A CA using transient expression in *Arabidopsis* mesophyll protoplasts. A schematic representation shows the effector, reporter and internal control plasmids used in the experiment. The reporter plasmids contained three tandem repeats of a 75-bp fragment of the *RD29A* promoter with DRE [1], the *RD29A* minimal promoter with a TATA sequence and the *GUS* reporter gene. To normalize the transfection efficiency and protoplast numbers, a plasmid containing a CaMV *35S* promoter-*ELuc* fusion gene was cotransfected as an internal control [2]. The values represent the average ratios of normalized GUS intensity relative to the intensity obtained with the empty effector plasmid; the error bars indicate SDs of triplicate technical repeats. Similar results were obtained in two independent experiments.
(TIF)

Figure S3 Proteasome inhibitors block the reduction of the DREB2A protein level after prolonged exposure to heat stress. Ten-day-old wild-type (WT) and *dreb2-1* seedlings were treated with 100 µM MG132 and exposed to heat stress (37°C). The level of DREB2A accumulation was determined by immunoblot analysis using the anti-DREB2A antibody. The arrowhead indicates the major band of DREB2A. The Rubisco large subunit (rbcL) bands visualized by Ponceau S are shown as loading controls.
(TIF)

Figure S4 MG115-induced accumulation of GFP-DREB2A is not sufficient for the induction of DREB2A target genes under normal conditions. Ten-day-old seedlings of *GFP/dreb2a* and two independent lines of *GFP-DREB2A/dreb2a* were treated with or without 200 µM MG115 under normal conditions (22°C) or conditions of heat stress (37°C). (A) Accumulation of GFP-DREB2A. The arrowhead indicates the major band of GFP-DREB2A in *GFP-DREB2A/dreb2a*. Each lane contains a total protein extract corresponding to 4 mg seedling FW. The rbcL bands visualized by Ponceau S are shown as loading controls. (B) The effects of MG115 treatment on the expression of DREB2A target genes. The mRNA levels of two heat-inducible DREB2A target genes were analyzed by RNA gel blot analysis using *DREB2A* cDNA and the cDNA of two DREB2A target genes *HsfA3* and *At4g36010*. Each lane contains 8 µg total RNA, and the rRNA bands (visualized by EtBr staining) are shown as loading controls. Similar results were obtained in two independent experiments.
(TIF)

Figure S5 Two-step model of the post-translational regulation of DREB2A involving stabilization and activation under stressful conditions. Under normal growth conditions, DREB2A is expressed at low levels. To minimize its activity when stresses are absent, DREB2A is ubiquitinated by the E3 ligases DRIP1/2 and subjected to proteolysis by the 26S proteasome. When subjected to dehydration and heat stress, the expression of *DREB2A* is enhanced via the *cis*-acting elements ABRE and HSE, respectively. At the post-translational level, stress signals stabilize DREB2A by inhibiting proteasome-dependent degradation. However, the accumulation of stabilized DREB2A is not sufficient for the induction of target genes. We propose that an additional activation step is required for the induction of target gene transcription. This activation step may involve a stress-specific modification or a protein-protein interaction.
(TIF)

Methods S1 Supporting methods.
(DOCX)
