# Supplementary figures and images for: Stabilization of Arabidopsis DREB2A Is Required but Not Sufficient for the Induction of Target Genes under Conditions of Stress
Source: PLoS One. 2013 Dec 23;8(12):e80457. doi: 10.1371/journal.pone.0080457 (PMC3871162; doi:10.1371/journal.pone.0080457)

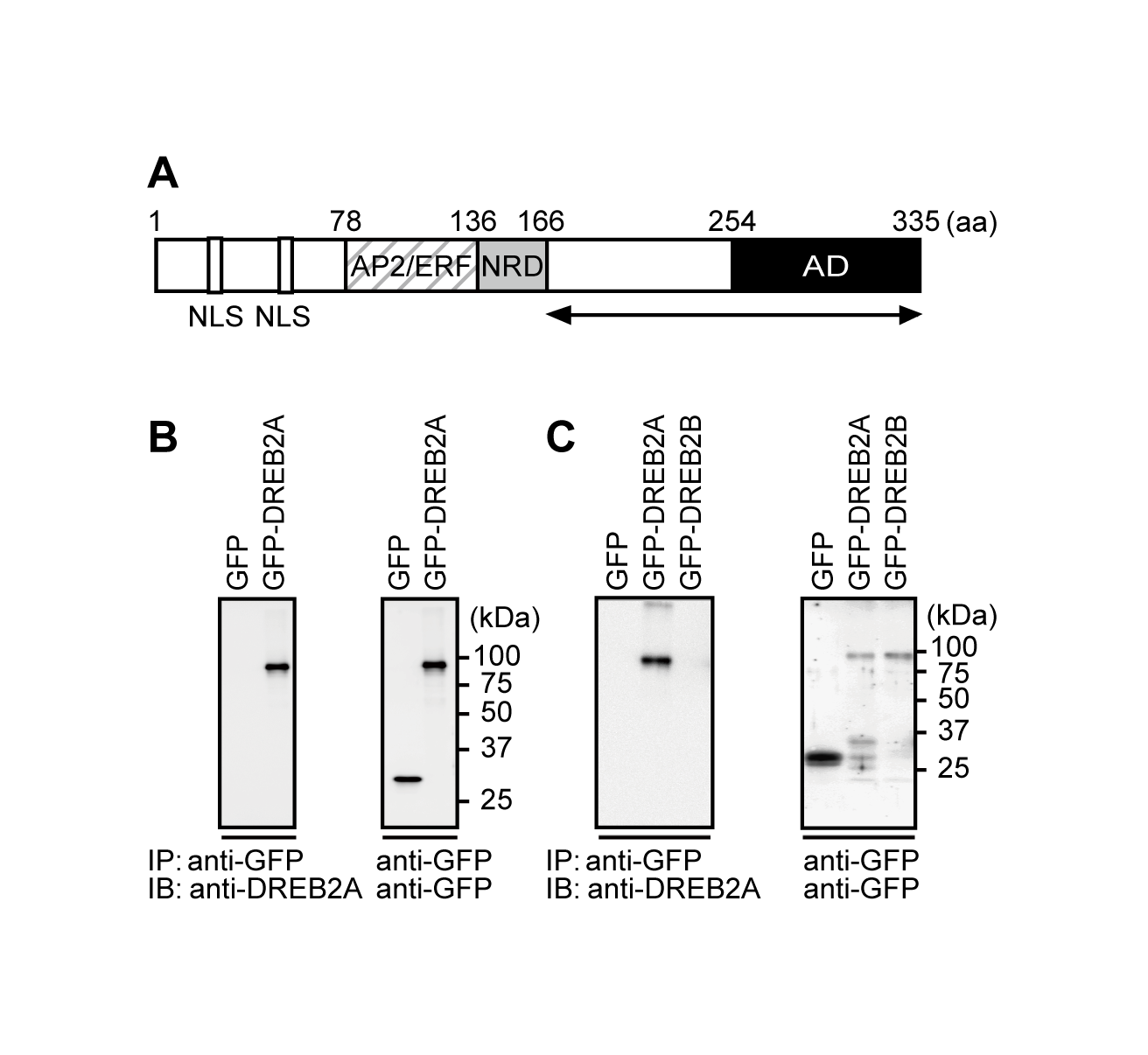

Supplement: Figure S1 — Specificity of the anti-DREB2A antibody. (A) Schematic representation of the DREB2A protein showing the region corresponding to amino acid residues 166–335 that was used to prepare the DREB2A polyclonal antibody (indicated by the double-headed arrow). NLS, nuclear localization signal; AP2/ERF, AP2/ERF DNA-binding domain; NRD, negative regulatory domain; AD, activation domain. (B) Evaluation of anti-DREB2A antibody activity. GFP or GFP-DREB2A was transiently expressed under the control of CaMV 35S promoter in Nicotiana benthamiana leaves and immunoprecipitated using anti-GFP microbeads. The precipitated fraction was analyzed by immunoblot with the anti-DREB2A antibody (left panel). The result of subsequent stripping and re-hybridization with anti-GFP antibody is shown in the right panel. (C) Confirmation of the specificity of the DREB2A antibody. GFP, GFP-DREB2A or GFP-DREB2B was transiently expressed under the control of the CaMV 35S promoter in N. benthamiana leaves and immunoprecipitated using anti-GFP microbeads. The immunoprecipitated fractions corresponding to 0.2 mg (GFP) or 2 mg (GFP-DREB2A and GFP-DREB2B) of leaves (FW) were loaded onto SDS-PAGE gels and analyzed by immunoblot with either the GFP (left panel) or DREB2A antibody. (TIF) [file pone.0080457.s001.tif]

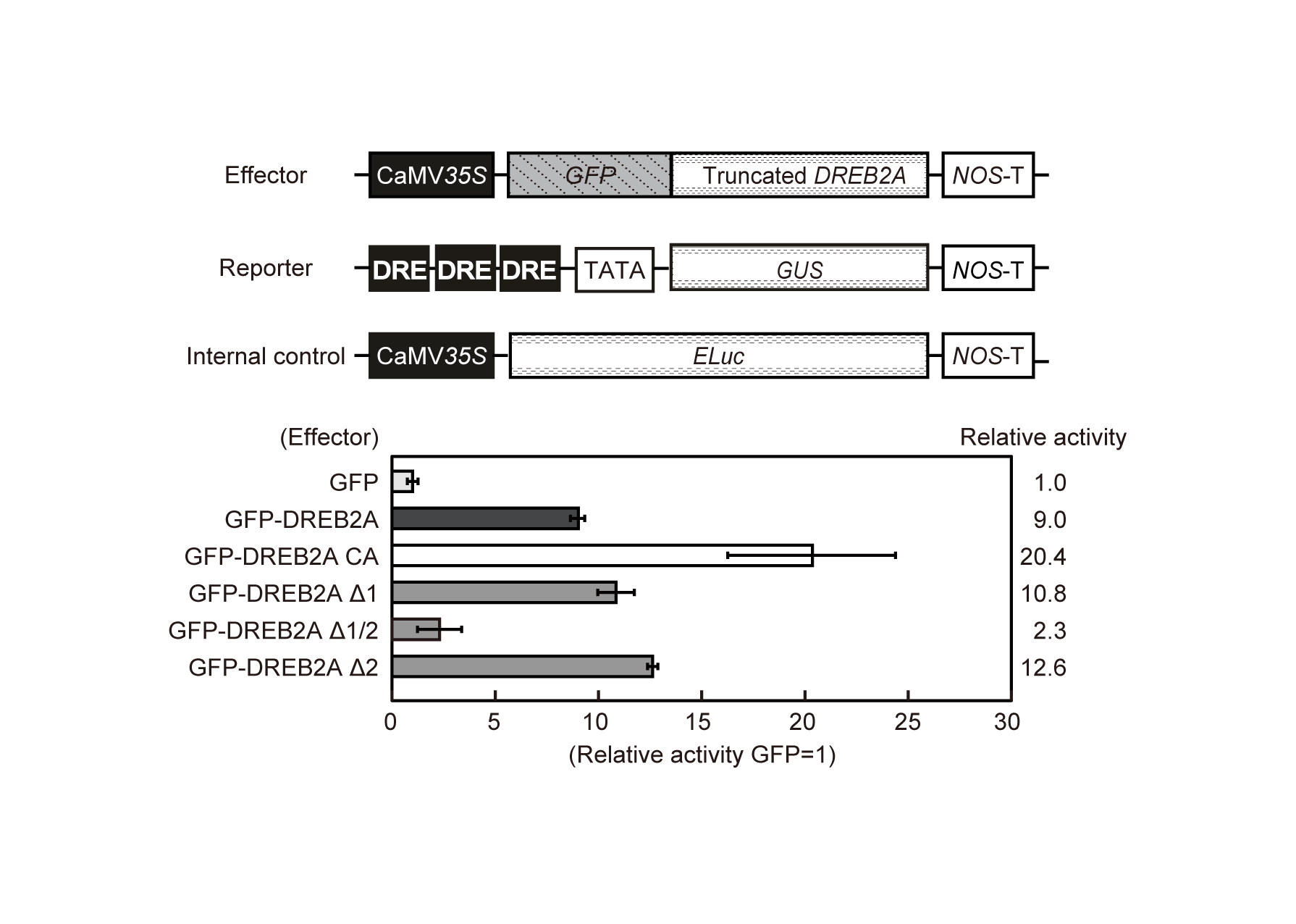

Supplement: Figure S2 — Transcriptional activity was retained in the GFP-DREB2A protein lacking one of the two nuclear localization signals (NLSs), but not in the protein lacking both of the NLSs. The transactivation activity of the GFP fusion proteins of DREB2A lacking one or both of the NLSs was compared with that of wild-type DREB2A and DREB2A CA using transient expression in Arabidopsis mesophyll protoplasts. A schematic representation shows the effector, reporter and internal control plasmids used in the experiment. The reporter plasmids contained three tandem repeats of a 75-bp fragment of the RD29A promoter with DRE [1], the RD29A minimal promoter with a TATA sequence and the GUS reporter gene. To normalize the transfection efficiency and protoplast numbers, a plasmid containing a CaMV 35S promoter-ELuc fusion gene was cotransfected as an internal control [2]. The values represent the average ratios of normalized GUS intensity relative to the intensity obtained with the empty effector plasmid; the error bars indicate SDs of triplicate technical repeats. Similar results were obtained in two independent experiments. (TIF) [file pone.0080457.s002.tif]

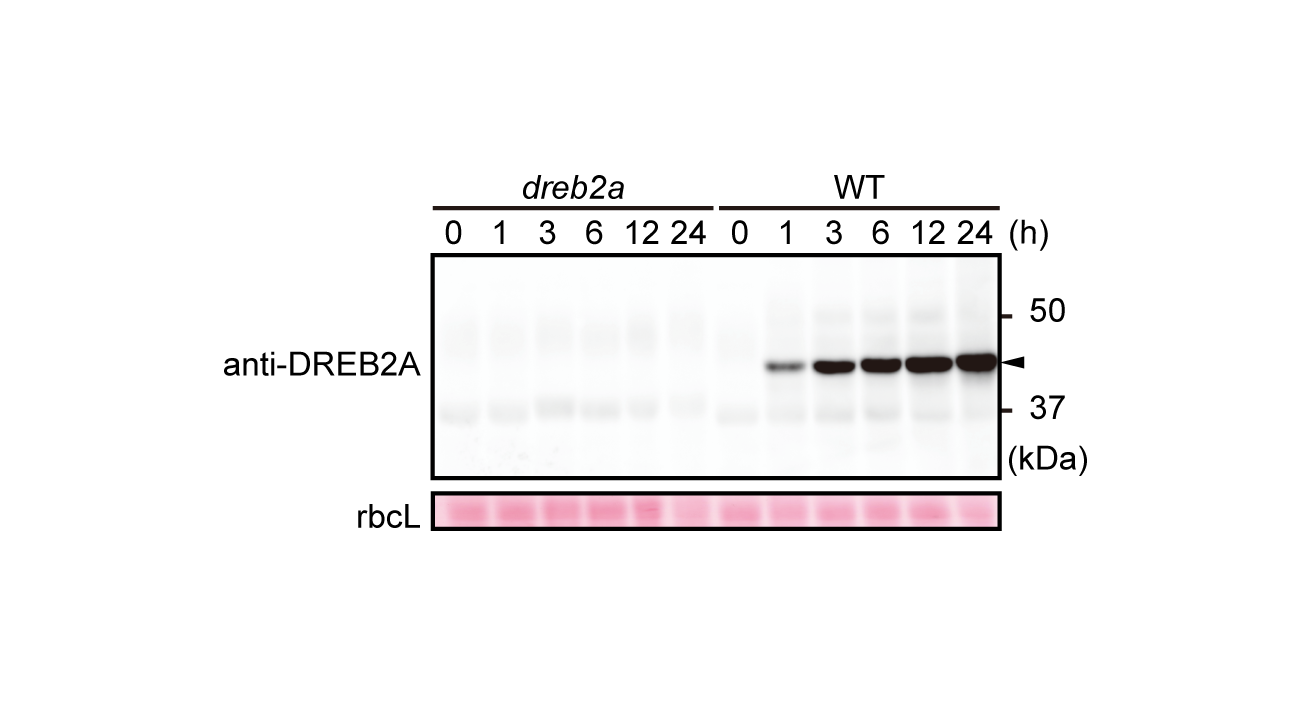

Supplement: Figure S3 — Proteasome inhibitors block the reduction of the DREB2A protein level after prolonged exposure to heat stress. Ten-day-old wild-type (WT) and dreb2-1 seedlings were treated with 100 µM MG132 and exposed to heat stress (37°C). The level of DREB2A accumulation was determined by immunoblot analysis using the anti-DREB2A antibody. The arrowhead indicates the major band of DREB2A. The Rubisco large subunit (rbcL) bands visualized by Ponceau S are shown as loading controls. (TIF) [file pone.0080457.s003.tif]

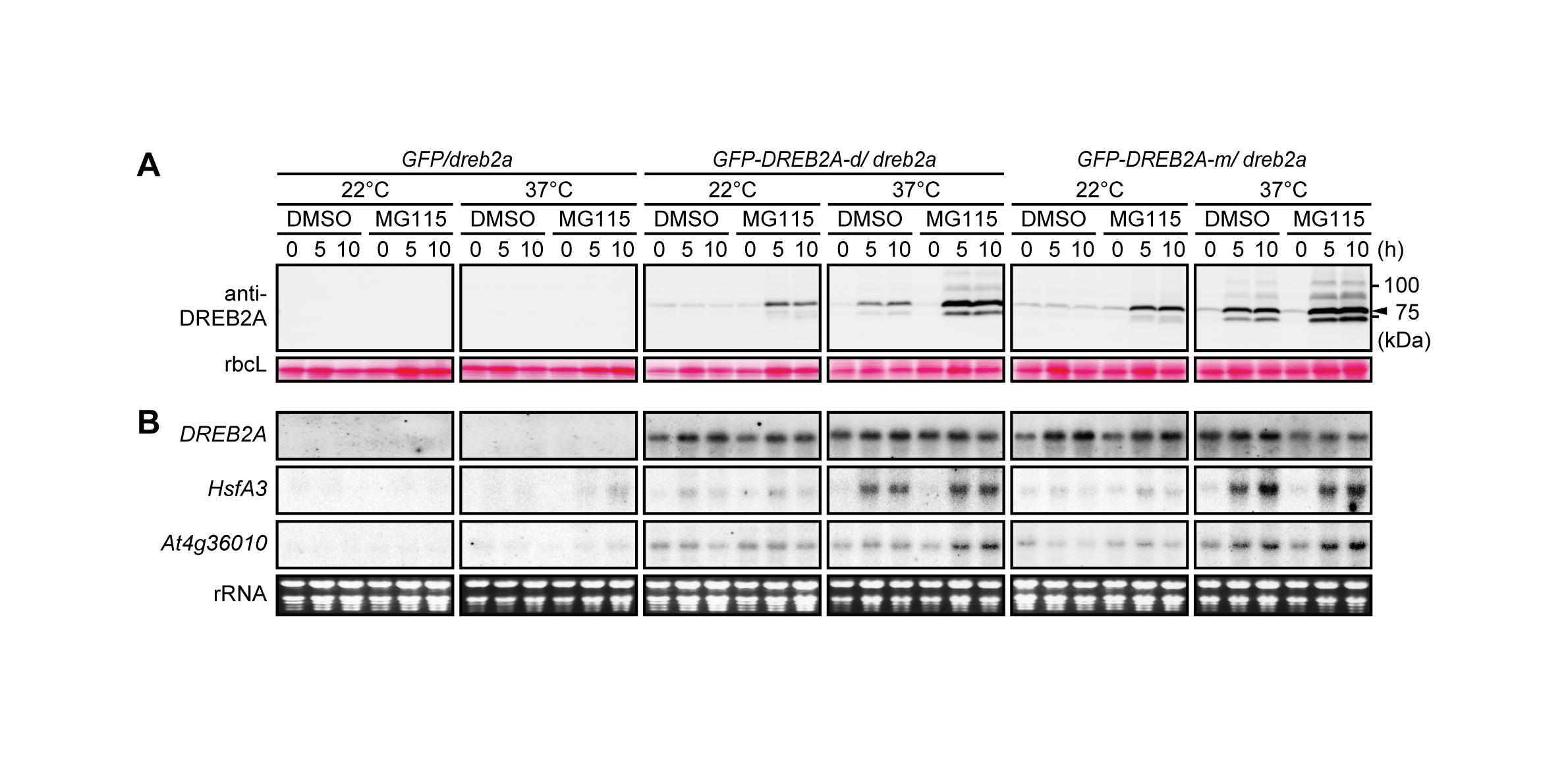

Supplement: Figure S4 — MG115-induced accumulation of GFP-DREB2A is not sufficient for the induction of DREB2A target genes under normal conditions. Ten-day-old seedlings of GFP/dreb2a and two independent lines of GFP-DREB2A/dreb2a were treated with or without 200 µM MG115 under normal conditions (22°C) or conditions of heat stress (37°C). (A) Accumulation of GFP-DREB2A. The arrowhead indicates the major band of GFP-DREB2A in GFP-DREB2A/dreb2a. Each lane contains a total protein extract corresponding to 4 mg seedling FW. The rbcL bands visualized by Ponceau S are shown as loading controls. (B) The effects of MG115 treatment on the expression of DREB2A target genes. The mRNA levels of two heat-inducible DREB2A target genes were analyzed by RNA gel blot analysis using DREB2A cDNA and the cDNA of two DREB2A target genes HsfA3 and At4g36010. Each lane contains 8 µg total RNA, and the rRNA bands (visualized by EtBr staining) are shown as loading controls. Similar results were obtained in two independent experiments. (TIF) [file pone.0080457.s004.tif]

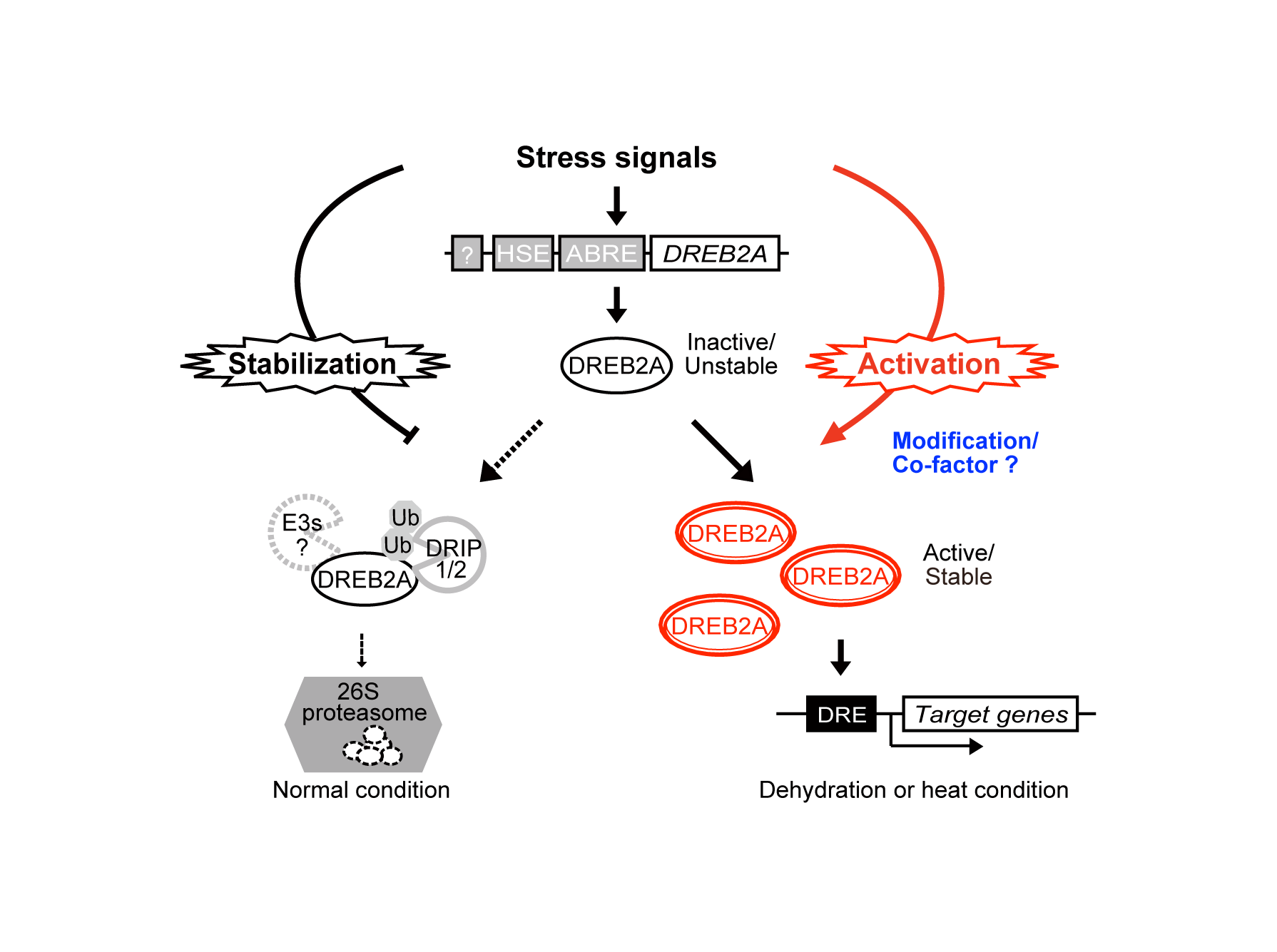

Supplement: Figure S5 — Two-step model of the post-translational regulation of DREB2A involving stabilization and activation under stressful conditions. Under normal growth conditions, DREB2A is expressed at low levels. To minimize its activity when stresses are absent, DREB2A is ubiquitinated by the E3 ligases DRIP1/2 and subjected to proteolysis by the 26S proteasome. When subjected to dehydration and heat stress, the expression of DREB2A is enhanced via the cis-acting elements ABRE and HSE, respectively. At the post-translational level, stress signals stabilize DREB2A by inhibiting proteasome-dependent degradation. However, the accumulation of stabilized DREB2A is not sufficient for the induction of target genes. We propose that an additional activation step is required for the induction of target gene transcription. This activation step may involve a stress-specific modification or a protein-protein interaction. (TIF) [file pone.0080457.s005.tif]
