# Supplementary material for: Stabilization of Arabidopsis DREB2A Is Required but Not Sufficient for the Induction of Target Genes under Conditions of Stress
Source: PLoS One. 2013 Dec 23;8(12):e80457. doi: 10.1371/journal.pone.0080457 (PMC3871162; doi:10.1371/journal.pone.0080457)
Supplement: Methods S1 — Supporting methods. (DOCX) [file pone.0080457.s006.docx]

**Methods S1**

Plasmid construction

To construct the plasmids used for the expression of the GFP or GFP-DREB2A fusion proteins in plants, an *sGFP* cDNA fragment [3] was amplified from pGFP3BX [4] by PCR using the primer pair 5’-GGGACTAGTATGGTGAGCAAGGGCGAG-3’ and 5’-GGGTCTAGATGCTTGTACAGCTCGTCCAT-3’. The *sGFP* cDNA fragment was inserted into the binary vector pBE2113Not [5] at the *Xba*I site. The resultant plasmid was named pBE2113_NsGFP. The entire coding sequence of *DREB2A* was amplified from the 35S-Ω-DREB2A plasmid [5] by PCR with the primer pair DREB2A/F-*Not*I (5’-AAGCGGCCGCATGGCAGTTTATGATCAGA-3’) and DREB2A/R-*Not*I (5’-AAGCGGCCGCGTTCTCCAGATCCAAGTAACT-3’). The amplified fragment was digested with *Eco*RV and cloned into pBE2113_NsGFP at the *Sma*I site in the sense orientation. The internal deletion mutants of the *DREB2A* cDNA were generated by inverse PCR with the 35S-Ω-DREB2A plasmid [5] followed by self-ligation. The primer pair DREB2A/54R (5’-CGATGTATCAATTTGTGTTCTG-3’) and DREB2A/79F (5’-GGTGACGGTACTACTGTGG-3’) was used to introduce the Δ1 mutation and the primer pair DREB2A/153R (5’-GGTAGAAACTTCTTCTACGGTC-3’) and DREB2A/166F (5’-GTACCTGCGAAAGGGTCG-3’) was used to generate the Δ2 mutation. The generated mutant fragments were subcloned into pBE2113_NsGFP as described above. To generate pGH-Histone2B-mRFP expressing the mRFP-Histone 2B fusion protein in *Arabidopsis* mesophyll protoplasts, the ORF of the monomeric RFP (mRFP) was amplified from a cDNA fragment (a gift from Dr. Roger Tsien, University of California, San Diego) with the primer pair mRFP/F-*Bam*HI (5’-GGATCCATGGTGGCCTCCTCCGA-3’) and mRFP/R-*Eco*RV (5’-GATATCCAGGAACAGGTGGTGGCGGCCCTC-3’). The amplified fragment was cloned between the *Bam*HI and *Eco*RV sites of pGreenII 0129 [6] with a CaMV *35S* promoter-nos terminator cassette transferred from the pGKX vector [7]. The resultant plasmid was named pG0129-35S:mRFP. The *Histone 2B* cDNA fragment was inserted between the *Xba*I and *Sma*I sites of pG0129-35S:mRFP to obtain pGH-Histone2B-mRFP.

Anti-DREB2A antibody

The anti-DREB2A antibody was produced in the rabbit using a His-tagged DREB2A fragment spanning amino acid residues 136-335 (DREB2A CT) that was expressed in *Escherichia coli* as an antigen. The coding sequence of DREB2A CT was amplified by PCR from a cDNA clone of DREB2A [5] using the primer pair DREB2A/406F-*Eco*RI (5’-AAAGAATTCCGGTCTGATGCGTCTGAG-3’) and DREB2A/C-*Sal*I (5’-AAAGTCGACTTAGTTCTCCAGATCCAAGT-3’). The amplified fragment was inserted between the *Eco*RI and *Sal*I sites of the pCold I expression vector (TaKaRa Bio). The resultant plasmid was named pCold-DREB2A CT and introduced into *E. coli* Rosetta (DE3) pLysS cells (Merck Millipore). The DREB2A CT protein expressed in the *E. coli* cells was purified using the Bug·Buster HisBind Purification Kit (Merck Millipore) according to the manufacturer’s instructions. Purified DREB2A CT protein was sent to Medical and Biological Laboratories (Nagoya, Japan) to be used for the immunization of a rabbit. The DREB2A antibody was purified as previously described [8] using a recombinant DREB2A protein spanning amino acid residues 166-335 (DREB2A 166-335). The coding sequence of DREB2A 166-335 was amplified by PCR using the primer pair DREB2A/496F-*Bam*HI (5’-GCGGATCCGATCCAGATTGTGAAT-3’) and DREB2A/C-*Sal*I and the resultant fragment was inserted between the *Bam*HI and *Sal*I sites of the pCold-GST-HRV3C vector [8]. The DREB2A 166-335 protein was expressed and purified as previously described [8].

References

[1] Liu Q, Kasuga M, Sakuma Y, Abe H, Miura S, et al. (1998) Two transcription factors, DREB1 and DREB2, with an EREBP/AP2 DNA binding domain separate two cellular signal transduction pathways in drought- and low-temperature-responsive gene expression, respectively, in Arabidopsis. Plant Cell 10: 1391-1406.

[2] Mizoi J, Ohori T, Moriwaki T, Kidokoro S, Todaka D, et al. (2013) GmDREB2A; 2, a Canonical DEHYDRATION-RESPONSIVE ELEMENT-BINDING PROTEIN2-Type Transcription Factor in Soybean, Is Posttranslationally Regulated and Mediates Dehydration-Responsive Element-Dependent Gene Expression. Plant physiol, 161: 346-361.

[3] Chiu W, Niwa Y, Zeng W, Hirano T, Kobayashi H, et al. (1996) Engineered GFP as a vital reporter in plants. Curr Biol 6: 325-330.

[4] Fujita M, Fujita Y, Maruyama K, Seki M, Hiratsu K, et al. (2004) A dehydration-induced NAC protein, RD26, is involved in a novel ABA-dependent stress-signaling pathway. Plant J 39: 863-876.

[5] Hellens RP, Edwards EA, Leyland NR, Bean S, Mullineaux PM, et al. (2000) pGreen: a versatile and flexible binary Ti vector for *Agrobacterium*-mediated plant transformation. Plant Mol Biol 42: 819-832.

[6] Qin F, Sakuma Y, Tran LS, Maruyama K, Kidokoro S, et al. (2008) Arabidopsis DREB2A-interacting proteins function as RING E3 ligases and negatively regulate plant drought stress-responsive gene expression. Plant Cell 20: 1693-1707.

[7] Kidokoro S, Maruyama K, Nakashima K, Imura Y, Narusaka Y, et al. (2009) The phytochrome-interacting factor PIF7 negatively regulates *DREB1* expression under circadian control in Arabidopsis. Plant Physiol 151: 2046-2057.

[8] Tanaka H, Osakabe Y, Katsura S, Mizuno S, Maruyama K, et al. (2012) Abiotic stress-inducible receptor-like kinases negatively control ABA signaling in Arabidopsis. Plant J 70: 599-613.
